# The Impact of Antenatal Corticosteroids on the Metabolome of Preterm Newborns: An Untargeted Approach

**DOI:** 10.3390/ijms25115860

**Published:** 2024-05-28

**Authors:** Enrico Valerio, Marta Meneghelli, Matteo Stocchero, Alfonso Galderisi, Silvia Visentin, Luca Bonadies, Paola Pirillo, Gabriele Poloniato, Giuseppe Giordano, Eugenio Baraldi

**Affiliations:** 1 Neonatal Intensive Care Unit, Department of Womens’ and Children’s Health, Azienda Ospedale-Università di Padova, 35128 Padova, Italy; marta.meneghelli@aopd.veneto.it (M.M.); matteo.stocchero@unipd.it (M.S.); luca.bonadies@aopd.veneto.it (L.B.); paola.pirillo@gmail.com (P.P.); poloniato.gabriele@gmail.com (G.P.); giuseppe.giordano@unipd.it (G.G.); eugenio.baraldi@unipd.it (E.B.); 2 Institute of Pediatric Research “Città della Speranza”, 35127 Padova, Italy; alfonsogalderisi@gmail.com; 3 Department of Pediatrics, Yale School of Medicine, Yale University, New Haven, CT 06511, USA; 4 Obstetrics and Gynecology, Department of Women’s and Children’s Health, University of Padova, 35128 Padova, Italy; silvia.visentin@unipd.it

**Keywords:** metabolomics, antenatal corticosteroids, prematurity, postnatal outcomes

## Abstract

We analyzed and compared variations in the urinary metabolome, as well as postnatal clinical outcomes among preterm infants, based on the timing of antenatal corticosteroid (ACS) administration in response to preterm labor onset in their mothers. This was a prospective observational study held in the Neonatal Intensive Care Unit, Department of Woman’s and Child’s Health, Padova University Hospital (Italy). A urine sample was obtained from each patient within 24 h of birth; Mass Spectrometry-based untargeted metabolomics analysis was then conducted. We searched for any significant disparities in the metabolomic profile of preterm newborns subjected to antenatal corticosteroid (ACS) treatment at varying timings; their correlation with clinical outcomes were also evaluated. The group receiving ACS within the optimal time window (1–7 days before delivery) exhibited elevated levels of cysteine, N-acetylglutamine, propionyl carnitine and 5-hydroxyindolacetic acid, coupled with a decrease in pipecolic acid. Clinically, this group demonstrated a reduced need for invasive ventilation (*p* = 0.04). In conclusion, metabolomics analysis identified several metabolites that discriminated preterm infants whose mothers received ACS within the recommended time window. Elevated levels of cysteine and 5-Hydroxyindoleacetic acid, metabolites characterized by antioxidant and anti-inflammatory properties, were observed in these infants. This metabolic profile correlated with improved respiratory outcomes, as evidenced by a reduced necessity for invasive ventilation at birth.

## 1. Introduction

Antenatal corticosteroids (ACSs) constitute the standard of care for pregnancies at risk of preterm delivery between 24- and 34-weeks gestational age (GA). The well-established benefits of ACSs in high-risk pregnancies within high-resource environments justify their administration, given their limited risk profile. Recently, however, possible harm including the risk of infectious disease morbidity throughout infancy and early childhood have recently been reported [1].

Different international guidelines provide recommendations on ACS treatment [2,3,4]; however, gaps exist in the comprehensive evaluation of ACSs’ benefits. This limitation arises from randomized controlled trials conducted prior to 1990, approximately, during a period when the standard of care for both mothers and preterm newborns significantly differed from contemporary practices. Furthermore, a substantial proportion of pregnancies included in these studies had gestational ages exceeding 28 weeks [5].

The optimal time interval between antenatal corticosteroid (ACS) treatment and delivery appears to be within 1 to 7 days. During this timeframe, maximal beneficial effects on fetal maturation are observed, resulting in the lowest odds of respiratory distress syndrome compared to both shorter and longer intervals between steroid administration and delivery [6]. Furthermore, critical considerations such as optimal ACS dosing, administration intervals, the number of courses, and pharmacokinetic and pharmacodynamic features remain unresolved [7,8]. Further investigations are warranted to delineate an individualized ACS treatment strategy that optimizes clinical responses while minimizing potential risks.

Metabolomics, the most recent of the -omics sciences, effectively fits within this modern context of care, which is increasingly moving towards individualized treatment of each patient. Metabolomic analysis of urine has already been applied in the neonatal field, particularly for the characterization of conditions such as sepsis [9], necrotizing enterocolitis [10], hypoxic-ischemic encephalopathy [11,12], and bronchopulmonary dysplasia [13]; however, there are currently no studies investigating the effect of antenatal steroid prophylaxis on the neonatal metabolome.

In this prospective observational study, untargeted metabolomics based on Mass Spectrometry (MS) was employed to investigate the urinary metabolome at birth in preterm infants requiring resuscitation in the delivery room, whose mothers received ACS due to the onset of preterm labor, with different time intervals relative to the actual delivery. Specifically, three groups were considered: one group of preterm newborns whose mothers completed a full course of ACS within 24 h to 7 days before delivery, another group with an incomplete course consisting of only one dose of ACS within 24 h of delivery, and a third group with one complete ACS course administered more than seven days before delivery. Additionally, we investigated whether postnatal clinical outcomes could be associated with differences in the urinary metabolome resulting from variations in the timing of ACS administration. To the best of our knowledge, this study is the first attempt to explore disparities in the urinary metabolome of preterm infants induced by different ACS administration timing.

## 2. Results

A total of 38 neonates requiring respiratory assistance at birth were enrolled, with assistance categorized as either non-invasive (delivered via face mask, *n* = 23) or invasive (endotracheal intubation, *n* = 15). Within this population, the group of neonates whose mothers received ACSs within the optimal time window (group O) consisted of 17 subjects. Meanwhile, the groups of neonates whose mothers received incomplete ACS treatment within 24 h of delivery (group I) and those whose mothers received ACSs more than 7 days before delivery (group C) comprised 11 and 10 subjects, respectively. The demographic and perinatal characteristics of the enrolled neonates are summarized in Table 1. No significant differences were observed among the three groups, except for a lower requirement for invasive mechanical ventilation among subjects in group O compared to the other two groups (*p* = 0.04).

Following the pre-processing of the metabolomics data, a dataset comprising 83 annotated variables and 38 observations was derived. No outliers were detected using the T2 test and Q-test, considering the PCA model for each individual group.

Given the potential influence of gestational age and the type of ventilation on the urinary metabolome, a correction was applied to the PLS-based model considering gestational age and type of ventilation as constraints in the model.

When comparing group I and group C, no differences were identified in the urinary metabolomic profile. Consequently, subjects from both groups were consolidated into a new single group, denoted as group I&C hereafter. The urinary metabolome of this combined group was then compared with that of the subjects in group O. Considering the type of ventilation, the *p*-value obtained from the chi-squared test with Yates correction was 0.03 when comparing group O to group I&C.

As an initial step, stability selection was employed to identify irrelevant variables. Subsequently, oCPLS2C was applied to the dataset, excluding the identified irrelevant variables. The resultant model, characterized by one predictive component and one orthogonal component, yielded a Matthew correlation coefficient of 0.63 (*p* = 0.045), with a cross-validated Matthew correlation coefficient of 0.52 (*p* = 0.002). A subset of eight relevant variables was discerned.

In MLR modelling, metabolite concentrations were regressed against gestational age, type of ventilation, and group. Four annotated variables were found to be significantly different between group O and group I&C.

**Figure 1:**
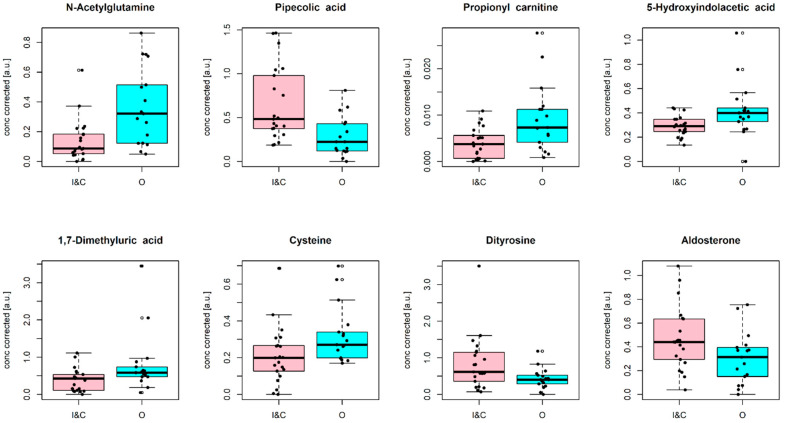
Boxplots illustrating the distributions of metabolite concentrations for the selected annotated variables, corrected for gestational age and type of ventilation. Pink, group I&C; turquoise, group O.

Merging the results of both multivariate and univariate data analysis, the following metabolites were identified as relevant: N-acetylglutamine, pipecolic acid, propionyl carnitine, 5-hydroxyindolacetic acid, 1,7-dimethyluric acid, cysteine, dityrosine, and aldosterone. Figure 1 depicts the distributions of the selected metabolites in the two investigated groups, represented as boxplots.

## 3. Discussion

Antenatal corticosteroids (ACSs) stand as one of the most effective interventions in perinatal medicine, significantly reducing neonatal mortality and improving severe outcomes associated with prematurity. ACS effectiveness extends to lowering the incidence of respiratory distress syndrome (RDS), the need for mechanical ventilation, as well as reducing the occurrences of intraventricular hemorrhage (IVH), periventricular leukomalacia (PVL), and mortality [14]. Newborns in the group treated within the optimal time frame exhibited a significantly reduced need for invasive ventilation (*p* = 0.04). This clinical observation suggests that the week preceding delivery is the optimal time interval to administer ACSs, yielding maximum beneficial effects on fetal lung maturation.

The mechanisms through which ACSs induce lung maturation remain incompletely understood, underscoring the necessity to enhance our understanding in this area. This study seeks to contribute to this knowledge gap, with the goal of identifying biomarkers that can offer insights into the efficacy of treatment and the associated biological pathways.

The application of metabolomics in this endeavor aligns with the paradigm of precision medicine [15,16]. In the midst of this transformative shift from standardized to personalized protocols, metabolomics holds the potential to play a pivotal role in optimizing antenatal treatments, including ACSs, to achieve the most favorable post-natal outcomes tailored to the individual patient.

In the neonatal subgroup whose mothers received ACSs within the optimal time interval (group O), notable differences were observed in comparison to those not receiving treatment during the optimal time (group I&C). Group O exhibited elevated levels of cysteine, N-acetylglutamine, propionyl carnitine, and 5-hydroxyindolacetic acid, while displaying lower concentrations of pipecolic acid and dityrosine compared to group I&C.

On the basis of our untargeted metabolomics analysis, none of these differences can be associated with differences in renal function.

Cysteine, elevated in group O, is one of the major intermediate products of cellular amino-acid metabolism. As a semi-essential amino acid, cysteine plays a crucial role in regulation of key endogenous antioxidant and anti-inflammatory pathways.

Reduced levels of cysteine have been documented in cardiovascular and neurological diseases, diabetes, and renal dysfunction [17,18,19].

Systemic inflammation and the generation of reactive oxygen species pose challenges in critically ill neonates, who frequently exhibit inadequate antioxidant systems. Pro-inflammatory conditions such as RDS, chronic lung disease, and perinatal brain injury are recognized as risk factors contributing to both short-term morbidity and long-term adverse neurodevelopmental outcomes [20,21,22,23,24]. Our findings indicate that the administration of ACSs within the optimal time window is associated with elevated levels of cysteine, which exerts known antioxidant and anti-inflammatory properties. This observation, along with potentially undiscovered effectors, suggests a potential link between appropriately timed ACSs and the inhibition of inflammasomes. Inflammasomes are defined as a family of multiprotein complexes [25,26,27] with the capability of inducing inflammatory cell death (‘pyroptosis’) [28] in the chorioamniotic membranes, which, in turn, may trigger preterm labor and delivery [29]. Specific inflammasome subtypes, such as NLRP3 [30] have been related to adverse neonatal outcomes, including bronchopulmonary dysplasia [31]. Conversely, the inhibition of NLRP3 has been associated with the prevention of preterm labor and delivery, as well as the improvement of outcomes linked to prematurity [32]. In this context, the well-established antioxidant and anti-inflammatory role of cysteine [33] may play a protective role in such infants, providing biologic plausibility to our results.

We observed elevated levels of N-acetylglutamine in group O compared to group I&C. N-acetylglutamine is a biologically available form of L-glutamine, the most abundant and versatile amino acid in the body, serving as the primary fuel for numerous cells. In critical illnesses, intense immune cell activity may lead to decreased plasma glutamine concentrations. This amino acid contributes nitrogen atoms crucial for the synthesis of purines, pyrimidines, and amino sugars, playing a pivotal role in cellular anabolism [34,35]; hence, a decrease in plasma glutamine levels may be indicative of inadequate glutamine production in skeletal muscle, excessive consumption by utilizing cells, or both [36]. Speculatively, the higher urinary level of this metabolite in group O could signify a lower metabolic demand, potentially attributable to reduced catabolism.

We found elevated levels of propionyl carnitine in the urine of group O. Propionyl carnitine is an acylcarnitine serving as a transporter of organic and fatty acids from the cytoplasm into the mitochondria where they undergo beta-oxidation to generate energy. In cardiovascular diseases, propionyl-L-carnitine has been recognized for its protective effects on the ischemic/reperfused heart. This protection is attributed to propionate’s ability to replenish mitochondria with dicarboxylic acid intermediates of the citric acid cycle and to increase cellular carnitine content, enhancing energy generation during the post-ischemic reperfusion phase [37].

The observed increase in the urine levels of propionyl carnitine in Group O may suggest heightened energy production. Notably, supplementation of short-chain carnitines has been investigated as potentially useful in various diseases, including neurological disorders and inborn errors of metabolism [38].

5-Hydroxyindoleacetic acid (5-HIAA) is the primary metabolite of serotonin. 5-HIAA starts as tryptophan within cells, which then converts to serotonin. Subsequently, monoamine oxidase A (MAO-a) enzymatically deactivates serotonin, transforming it to 5-HIAA within the synaptic cleft. Diseases or disorders associated with an increase in urinary 5-HIAA include intestinal neuroendocrine tumors, celiac disease, cystic fibrosis, and autism spectrum disorder [39].

Recent studies have revealed a positive correlation between 5-Hydroxyindoleacetic acid (5-HIAA) levels and lung function, alongside a negative correlation with exhaled nitric oxide (NO) in asthmatic patients [40]. Intriguingly, our observation of elevated levels of this metabolite in the urine of infants in group O prompts speculation regarding the potential long-term respiratory benefits associated with the optimal timing of ACS administration in these patients.

Pipecolic acid, a downstream catabolite of lysine [41], exhibited lower concentrations in infants receiving ACSs between 24 h and 7 days before birth. The diminished concentration in the urine of newborns receiving ACSs within the correct time frame suggests a reduction in lysine catabolism in this patient group. These findings align with the recognized neuroprotective and neuromodulatory role of intact lysine in animal models of brain damage [42,43] and may, in part, elucidate the observed link between well-timed ACS administration and improved neurological and overall outcomes in this class of patients, as reported in the literature [6].

To the best of our knowledge, this exploratory study is the first attempt to apply untargeted metabolomics to a population of resuscitated preterm infants, whose mothers received ACSs at varying time intervals relative to delivery, in order to discern differences in their postnatal urinary metabolomic profiles.

Recent studies in the literature focus on neonatal steroid profiling [44]; in our study, we did not perform specific steroid profiling because we considered a larger set of metabolites.

The contradiction inherent in the fact that the metabolomic effects of antenatal steroid prophylaxis do not directly involve steroid molecules in our results is, in any case, apparent; indeed, the metabolic effects of antenatal steroid administration at different timings have been poorly studied in the literature and are likely mediated by a series of effector molecules (e.g., cysteine, which plays a regulatory role towards antioxidant and anti-inflammatory pathways) not necessarily directly correlated with the steroid nature of maternal prophylaxis.

One limitation of this study is the absence of a group of preterm infants who did not receive ACS prophylaxis. Given the recognized benefits of ACS administration, almost all women at risk of preterm delivery now receive this treatment. Another limitation pertains to the small number of patients included in this study.

## 4. Materials and Methods

### 4.1. Experimental Design

This prospective observational study included singleton preterm newborns with a gestational ranging from 23 to 34 weeks, who necessitated respiratory resuscitation in the delivery room. Participants were admitted to the Neonatal Intensive Care Unit (NICU) within the Department of Woman’s and Child’s Health at Padova University Hospital, Italy, from April 2015 to September 2019.

Exclusion criteria comprised infants with major congenital abnormalities or chromosomal abnormalities, known or suspected congenital metabolic diseases, those experiencing asphyxia, newborns who had received transfusions before urine collection, and those for whom consent was refused.

A single course of two doses (12 mg per dose) consisting of a 1:1 mixture of betamethasone phosphate and betamethasone acetate, separated by 24 h, is the recommended treatment for mothers at risk of preterm delivery [2].

We classified the cases into three groups, as follows: group O (Optimal): one complete course of ACS, with a time window from treatment to delivery of 24 h to 7 days, as per the optimal time interval suggested in the literature [6]; group I (Incomplete): an incomplete course involving only one dose of ACS administered within 24 h before delivery; and group C: one complete ACS course administered more than 7 days before delivery.

All pertinent clinical data during the Neonatal Intensive Care Unit (NICU) stay were systematically recorded for each enrolled subject (Table 1). A urine sample was collected from each subject within 24 h of life. This study received approval from the Hospital Ethics Committee (Reference Number 3636/AO/15), and informed consent was obtained from the parents of each patient before their admission to the study.

### 4.2. Sample Collection

Noninvasive collection of at least 2 mL of urine was performed for each subject by placing a sterile cotton ball inside the newborn’s diaper and checking for the presence of urine every 30 min until 24 h of life, as previously described [45].

### 4.3. Metabolomic Analysis

Mass Spectrometry (MS)-based untargeted metabolomics analysis of urine was conducted in both positive and negative ionization mode using an Acquity ultra performance liquid chromatography (UPLC) system (Waters MS Technologies, Ltd., Manchester, UK) coupled to a quadrupole time-of-flight (QToF) Synapt G2 HDMS mass spectrometer (Waters MS Technologies, Ltd., Manchester, UK). Chromatography was performed using an Acquity HSS T3 (1.7 μm, 2.1 × 100 mm) column (Waters Corporation, Milford, CT, USA) maintained at 50 °C. Data acquisition and chromatographic settings were the same as described in the reference [45].

Standard solution samples and quality controls (QCs) were incorporated to assess reproducibility and accuracy during the analysis. Specifically, QCs with three different dilution factors (1:2, 1:3 and 1:5), obtained by pooling the collected samples and using a 0.1% formic acid solution for dilution, were used. Data extraction was performed using Progenesis QI software (Waters Corporation, Milford, CT, USA), with parameters optimized through preliminary analysis of the QCs. The intensities of the extracted features were standardized by calculating local calibration models using the QCs, as previously outlined [46]. Probabilistic quotient normalization was employed to mitigate the effects of sample dilution, and any variables with a coefficient of variation in the QCs exceeding 20% were excluded. Variables were annotated after searching our in-house database, resulting in a level of annotation equal to 1 for the annotated variables [47]. The data pertaining to annotated variables were autoscaled and submitted to data analysis.

### 4.4. Statistical Data Analysis

Demographic and perinatal characteristics of the recruited neonates were examined using the chi-squared test for categorical data and the Kruskal–Wallis test for numerical data.

Both multivariate and univariate analyses were conducted. Specifically, Principal Component Analysis (PCA) was used for exploratory data analysis and outlier detection, whereas orthogonally constrained PLS for classification (oCPLS2C) was employed for comparing the groups under investigation [48,49]. Stability selection based on variable influence on projection was performed to discover relevant and irrelevant variables [50]. The reliability of the model was evaluated through repeated 5-fold full cross-validation with 20 repetitions, accompanied by a permutation test on the class response involving 1000 random permutations.

For univariate data analysis, metabolite concentrations were modelled using Multiple Linear Regression (MLR). A significance level of 0.05 was adopted in the data analysis.

All data analyses were conducted using in-house R-functions implemented on the R 4.0.4 platform (R Foundation for Statistical Computing).

## 5. Conclusions

This study highlights the fact that the metabolome of preterm infants whose mothers received ACS in the recommended time window is enriched in cysteine and 5-Hydroxyindoleacetic acid—metabolites known for their antioxidant and anti-inflammatory properties. Furthermore, the observed decrease in pipecolic acid, a downstream catabolite of lysine, aligns with a potential neuroprotective effect. From a clinical point of view, the group with optimal ACS timing showed a better respiratory outcome, with less need of invasive ventilation at birth. This study must be considered a hypothesis-generating, exploratory study; future research endeavors will be essential to elucidate the intricate biological roles of these metabolites in enhancing outcomes for preterm infants undergoing ACS treatment.

## Figures and Tables

**Table 1:** Characteristics of the recruited neonates: categorical data are expressed as the number of cases, while numerical data are expressed as the median [IQR]; *p* is the *p*-value of the statistical test. IQR, interquartile range; EOS, early-onset sepsis; LOS, late-onset sepsis.

Characteristics	Group O *n* = 17	Group I *n* = 11	Group C *n* = 10	*p*
Sex, male (female)	6 (11)	5 (6)	4 (6)	0.87
Gestational age [weeks.days], median (IQR)	29.1 (4.1)	27.1 (5.1)	28.2 (4.6)	0.95
Birth weight [g], median (IQR)	1050 (545)	1075 (558)	1285 (736)	0.58
Delivery mode, vaginal (caesarean section)	1 (16)	2 (9)	1 (9)	0.58
Apgar 1 min, median (IQR)	6 (3)	4 (2)	6 (2)	0.18
Apgar 5 min, median (IQR)	8 (1)	7 (2)	7 (0.8)	0.13
Apgar 10 min, median (IQR)	8 (1)	8 (0.5)	8 (1.8)	0.22
Ventilatory support, non-invasive (invasive)	14 (3)	4 (7)	5 (5)	0.04
Chorioamnionitis, yes (no)	7 (10)	2 (9)	6 (4)	0.14
Intrauterine growth restriction, yes (no)	4 (13)	1 (10)	0 (10)	0.19
Sepsis, EOS (LOS) [no]	3 (5) [9]	3 (4) [4]	2 (3) [5]	0.94
Necrotizing enterocolitis, yes (no)	0 (17)	1 (10)	3 (7)	0.05
Intraventricular hemorrhage, yes (no)	0 (17)	2 (9)	1 (9)	0.21
Respiratory distress syndrome, yes (no)	8 (9)	9 (2)	6 (4)	0.19
Bronchopulmonary dysplasia 28 days, yes (no)	7 (10)	6 (5)	4 (6)	0.74
Bronchopulmonary dysplasia 36 weeks, yes (no)	5 (12)	3 (8)	3 (7)	0.99
Retinopathy of prematurity, yes (no)	5 (12)	4 (7)	3 (7)	0.92
Death, yes (no)	0 (17)	0 (11)	0 (10)	1.00

## Data Availability

The data presented in this study are available on request from the corresponding author.
